# Entropy and Network Centralities as Intelligent Tools for the Investigation of Terrorist Organizations

**DOI:** 10.3390/e23101334

**Published:** 2021-10-13

**Authors:** Alexandros Z. Spyropoulos, Charalampos Bratsas, Georgios C. Makris, Evangelos Ioannidis, Vassilis Tsiantos, Ioannis Antoniou

**Affiliations:** 1 Department of Physics, School of Science, Kavala’s Campus, International Hellenic University (IHU), 57001 Thessaloniki, Greece; tsianto@physics.ihu.gr; 2 Department of Mathematics, Aristotle University of Thessaloniki (AUTH), 54124 Thessaloniki, Greece; cbratsas@math.auth.gr (C.B.); geomak@auth.gr (G.C.M.); ioannidek@math.auth.gr (E.I.); iantonio@math.auth.gr (I.A.)

**Keywords:** terrorist networks, police investigations, criminal investigations, centralities measures, entropy in crime investigation, weighted network, network roles identification

## Abstract

In recent years, law enforcement authorities have increasingly used mathematical tools to support criminal investigations, such as those related to terrorism. In this work, two relevant questions are discussed: “How can the different roles of members of a terrorist organization be recognized?” and “are there early signs of impending terrorist acts?” These questions are addressed using the tools of entropy and network theory, more specifically centralities (degree, betweenness, clustering) and their entropies. These tools were applied to data (physical contacts) of four real terrorist networks from different countries. The different roles of the members are clearly recognized from the values of the selected centralities. An early sign of impending terrorist acts is the evolutionary pattern of the values of the entropies of the selected centralities. These results have been confirmed in all four terrorist networks. The conclusion is expected to be useful to law enforcement authorities to identify the roles of the members of terrorist organizations as the members with high centrality and to anticipate when a terrorist attack is imminent, by observing the evolution of the entropies of the centralities.

## 1. Introduction

Law enforcement authorities (police-judiciary), which are entrusted with the tasks of preventing and detecting terrorist attacks, are particularly interested in effective methods of identifying the different roles of the members of a terrorist organization. The presence of different roles (division of tasks) within a terrorist organization is one of the four basic conditions for an organization to be classified as a terrorist/criminal organization, as accepted for example by the members of the European Union [1,2]. As terrorist acts are a real threat to the modern societies [3], the authorities need also effective methods to diagnose imminent terrorist attacks from early signs, so that the necessary preventive actions can be taken. The goal of this work is to apply the tools of *entropy* and *network theory* to address the above problems.

Network theory studies the geometric representation of the relations between the components of a system as graphs [4,5,6,7,8,9,10,11,12]. The components of a system are represented by nodes and the relations between the nodes by links [6,10,13,14,15].

Many natural or artificial systems are modelled as networks: computers, telecommunications, water supply, transport, power chains, organs of the human body or cellular interaction, protein networks, networks of meteorological phenomena. Social networks in particular represent the relationships between people [10,16].

Networks are directed or undirected (symmetric) depending on the nature of the links. Telecommunication networks are directed if the calls are characterized as outgoing or incoming [8,9,10,17]. If only the presence of contacts between people is relevant, the communication network is undirected, as in the case of law enforcement investigations [18,19]. Links may be weighted according to the modelling requirements. For example the relationship of two people who have met 20 times in a given period of time should have more weight compared to the link between two people who have met only once [7,10,20,21].

Law enforcement authorities have understood that modern crime is a complex system, as the motivations of human behaviours, as well as the factors of upbringing and development of each person, vary [22,23,24,25,26]. Nevertheless, the modelling of criminal behaviours involves the analysis of the spatio-temporal patterns of the commission of the crimes [23,27]. This fact is the reason that network theory is useful for the drawing inferences on anti-legislative actions and policies [18,19,20,21,23,28,29,30,31,32,33,34,35,36,37].

The construction of networks for monitoring by law enforcement authorities can be done either by visualizing telecommunication networks or by recording human encounters—relationships. Telecommunication networks are created with weight (as many times as the communication times of the nodes) and direction (incoming-outgoing communication). Physical contact networks are created with weight (the number of joint meetings) but without direction. International practice seems to reach a consensus that the conclusions drawn from the analysis of personal contact networks appear to be more reliable than those drawn from telecommunication contacts networks [18,19].

Network theory provides the tools to highlight the roles of nodes resulting from the relations to the other nodes of the network [6,7,8,10,11,17,28,38,39,40,41,42,43,44], by computing selected local statistical indices known as *centralities*. This is particularly applicable to the analysis of delinquent networks by the law enforcement authorities [10,28,38,40,42,43]. In this first exploratory study, three key node properties of interest to law enforcement authorities are selected, namely, (1) the number of direct contacts of each member of the network, because members with many contacts contribute more in the network and in this sense, they are more “useful”; (2) the “liaison officers” in a communication network mediate in the communication pathways among the members of the network. Nodes patriating in many communication pathways are good mediators coordinating the network activities and their elimination results in limitations of the network operations; (3) If the neighbours of a member, contact each other, then this member is probably a team leader. Such “teamworking nodes” serve as nuclei of small operating teams and their elimination results in disabling their team. These three key properties are assessed quantitatively by the corresponding centralities, namely, the degree centrality, the betweenness centrality and the clustering coefficient.

As *entropy* is a global measure of the diversification of any system, the competent authorities have already used entropy as a tool for analysing criminal networks [45,46] and as an assessment of relationships between different terrorist networks [47]. Entropy has also recently been used to predict criminal behaviours [48,49,50]. Prediction involves the study of temporal networks [51,52], of particular interest in criminal investigations, because the role and/or effectiveness of the nodes usually changes in time [28,29,33,34,47,53]. High entropy of some local network properly indicates that many nodes share this property in more or less the same degree.

The goal of this work is to address the above problems, with the tools of entropy and network theory. More specifically, the following questions arise which are expected to enhance the capabilities of law enforcement authorities:

Q1: “How can the different roles of members of a terrorist organization be recognized?”

Q2: “Are there early signs of impending terrorist acts?”

The research methodology (centralities and their entropies) is presented in Section 2 and applied to data (Section 3) of four real terrorist networks from different countries. The results are presented in Section 4 and discussed in Section 5.

## 2. Methodology

The research questions Q1 and Q2 are addressed using selected tools of network theory, applied to the physical contact networks of four real terrorist organizations, from different countries. The selected tools of network theory are three centralities (degree, betweenness, clustering) to address Q1 and the corresponding entropies to address Q2. The centralities and entropies are described below.

### 2.1. Centrality Measures

*Centralities* are measures indicating the importance of each node, resulting from the topology of links [8,10,17,40,41,54,55]. The importance of nodes is assessed by ranked the nodes according to the values of their centralities. There are more than 100 such indicators that refer locally to each node. From centralities, global indicators are computed like centralizations, averages and entropies showing an overall assessment of the network [10,11,17]. In this article, selected local indicators are examined for undirected networks, namely, degree centrality, betweenness centrality and clustering coefficient:

#### 2.1.1. Degree Centrality

The degree of node i in a network of order N is the number of connections of the node i and takes values from 0 to N−1. The value 0 indicates the absence of links and there are no self-loops. The normalized degree is the degree centrality [10,38]: $DEG_{\kappa }=\frac{\sum_{\lambda =1}^{N}a_{\kappa \lambda }}{N-1}$, where: $a_{\kappa \lambda }$ is the κλ–element of the adjacency matrix [5,6,7,11] of the network. In the case of weighted networks, the weighted degree is known as strength: $DEG_{\kappa }^{\left[w\right]}=\frac{\sum_{\lambda =1}^{N}w_{\kappa \lambda }}{N-1}$. 

#### 2.1.2. Betweenness Centrality

The betweenness of a node indicates how influential the node is by judging whether the node in question lies within the path joining pairs of other nodes [10,38] and takes values from 0 to $\left(N-1\right)\left(N-2\right)$. The betweenness centrality of node κ is defined by the formula:

$B_{\kappa }=\frac{1}{\left(N-1\right)\left(N-2\right)}\overset{N}{\underset{\begin{array}{c} \kappa ,\lambda ,\mu =1\\ \kappa \;\neq \;\lambda \;\neq \;\mu \end{array}}{\sum }}\frac{\sigma_{\lambda \left(\kappa \right)\mu }}{\sigma_{\lambda \mu }}$, where $\sigma_{\lambda \mu }$ is the number of paths connecting nodes λ and μ and $\sigma_{\lambda \left(\kappa \right)\mu }$ is the number of paths connecting nodes λ and μ and passing through the node κ. 

#### 2.1.3. Clustering Coefficient 

The neighbourhood density of a node indicates the extent to which its first neighbours are linked to each other. The neighbourhood density of node i, also known as clustering coefficient of node κ [11], is calculated from the formula: $clu_{\kappa }=\frac{2E_{\kappa }}{{𝓋}_{\kappa }\left({𝓋}_{\kappa \;}-1\right)}$, where $E_{\kappa }$ is the number of links between the first neighbours of node κ, and ${𝓋}_{\kappa }$ is the number of first neighbours of node κ.

### 2.2. Identification of Roles

The role of nodes of the network according to the selected relevant criteria is assessed by the values of the corresponding centralities. For example, in the cooperation network of the employees of a company, the nodes with *high degree* are the popular employees or the employees with many responsibilities. Betweenness centrality identifies the employees who act as *mediators* between different employees. The *team players* or *teamworking nodes* are the employees with high clustering coefficient [10,28,38,40,42,43].

### 2.3. Entropy of Centralities

Entropy of some random variable X is the average information obtained from the measurement of the n values $x_{1}$, $x_{2},\;$…, $x_{n}$ of a variable X. Therefore, the entropy is a measure of the lack of information before more accurate measurements are made. The Boltzmann, Planck, Gibbs’ entropy of statistical physics is [56,57]: $S^{BPG}\left[p\right]=-\overset{n}{\underset{i=1}{\sum }}p_{i}lnp_{i}$. In this work, Shannon’s entropy is used, representing the minimal average length of binary coding [58,59]:

$$S=-\overset{n}{\underset{i=1}{\sum }}p_{i}\log_{2}p_{i}$$

In order to compare the entropies of different variables the normalized entropy $\frac{S}{\log_{2}n}$ is computed, taking values in the interval $\left[0,1\right]$. Entropy is a measure of the diversity of the values of the variables. High entropy indicates that most values are more or else equally probable, while low entropy indicates that few values are highly probable and dominate, as the other values have rather low probability. Shannon’s formula is used to calculate the entropies of the three selected centralities, namely, degree, betweenness and clustering defined in Section 2.1. The *degree entropy* is: $S_{deg}=-\overset{N-1}{\underset{i=1}{\sum }}p_{i}\log_{2}p_{i}$, where $p_{i}$ is the probability distribution of the values of the degree centrality. The normalized degree entropy is $\frac{S_{deg}}{\log_{2}\left(N-1\right)}$. The *betweenness entropy* $S_{B}$ and the *clustering entropy* $S_{Clu}$ are defined in the same way. In the case of networks, centralities with high entropy indicate high diversification of roles of the nodes, while low entropy indicates that most nodes have lack roles in the network.

The additivity of Boltzmann, Planck, Gibbs’ entropy reflects non-extensivity of ergodic systems close to equilibrium. This is the standard assumption of most systems of statistical mechanics and information theory [12,59,60,61]. Other entropies like Renyi entropy have been proposed with interesting applications beyond Statistical physics [12]. The study of anomalous systems with long range interactions with metastable long lived states requires non-extensive entropies like Tsallis entropy [12,62]. The physical contact networks under investigation are assumed to be regular as there are no indications of anomalies.

## 3. Data Sets

Physical contact networks of four real terrorist organizations, from four different countries are selected. The data sets are in the public domain. The real names of the members of the organizations are encrypted. Finding such data sets is much more difficult, compared to other datasets as they are usually classified.

### 3.1. Terrorist Organization “Jamaah Islamiah Section of Indonesia”

The physical contacts [63] between the identified members of the Jamaah Islamiah terrorist organization were recorded by the Indonesian police from 1985 to 2007 [64]. The data include 11 time periods depicting 27 individuals (nodes). The network of physical contacts is fully connected (no disconnected nodes or groups of nodes), weighted and undirected.

### 3.2. Terrorist Organization “Hamburg Cell” 

The physical contacts [65] between the identified members of the Hamburg Cell terrorist organization were recorded by the German and United States authorities from 1985 to 2006 [66]. The data include 15 time periods depicting 34 individuals (nodes). The network of physical contacts includes isolated nodes and is weighted and undirected.

### 3.3. Terrorist Organization “Al-Qaeda Section of Madrid”

The physical contacts [67] between the identified members of the al-Qaeda section of Madrid were recorded on the occasion of the terrorist attack in 2004 and were recorded by the Spanish authorities from 1985 to 2006 [68]. The data include 14 time periods in which 54 people (nodes) are depicted. The network of physical contacts includes isolated nodes and is weighted and undirected.

### 3.4. Terrorist Organization “Jamaah Islamiah Section of Philippines”

The physical contacts [69] between the identified members of the Jamaah Islamiah section of Philippines were recorded on the occasion of the terrorist attack of 2000 and were recorded by the Spanish authorities from 1985 to 2006 [70]. The data represent 14 time periods in which 16 atoms (nodes) are displayed. The network of physical contacts is fully connected (no disconnected nodes or groups of nodes), weighted and undirected.

## 4. Results

From the above data, the three centralities (degree, betweenness, clustering) and the corresponding entropies are computed for the networks of the four terrorist organizations.

The values of centralities indicate that some nodes are stand out nodes. The results are presented below:

### 4.1. Terrorist Organization “Jamaah Islamiah Section of Indonesia”

**Figure 1:**
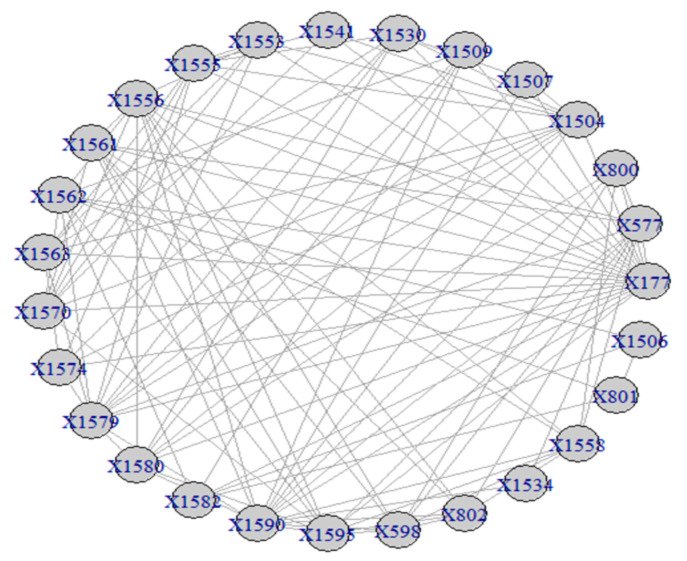
The network of “Jamaah Islamiah section of Indonesia”.

The visualization of the overall network over the period 1985–2007 is presented in Figure 1.

**Figure 2:**
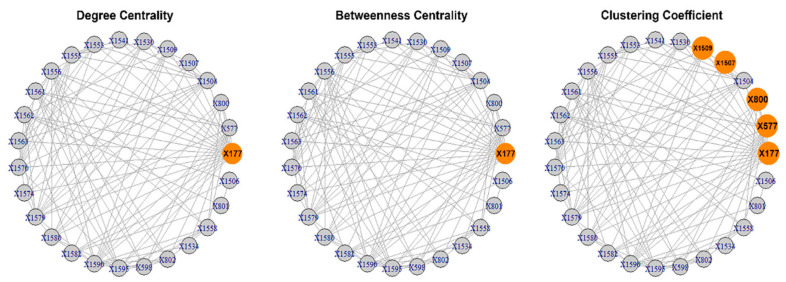
The overall protagonists of the “Jamaah Islamiah section of Indonesia”. The protagonists are represented as larger oranges spheres. Most nodes have degree >0.3, but the node 177 (0.7) stands out. Most nodes have low values (<0.2) as mediators, but the 0177 (0.3) stands out. Most nodes are team players (clustering >0.6), but the nodes 177 (1), 577 (1), 800 (1), 1504 (1), 1507 (1) and 1509 (1) stand out as team players.

**Figure 3:**
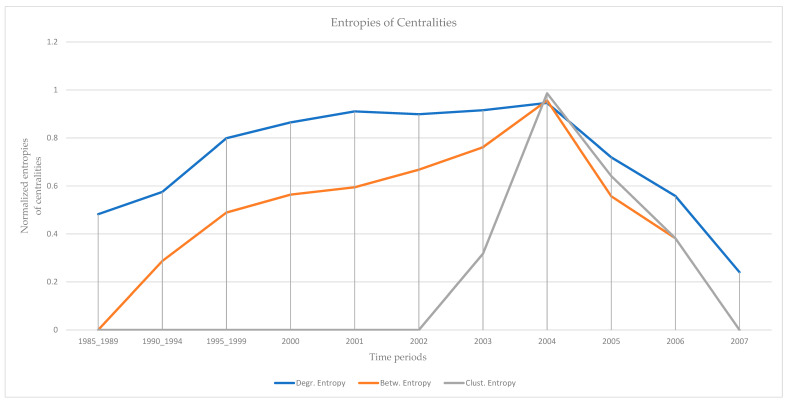
The evolution of normalized entropies. A peak of the entropies appears in 2004. The entropies increase from 1995 to 2004, but the entropy of clustering increases shakenly after 2002. All entropies decrease after 2004, the entropy of betweenness decreases a little faster.

The results of the calculations of the centralities and the entropies are found in Supplementary Material 1. From the values of the centralities, the *protagonists* (high centrality nodes) for the period 1985–2007 (overall) are identified and presented in Figure 2. The protagonists for each time period are presented in Table 1. The evolution of the entropies of the centralities is presented in Figure 3.

### 4.2. Terrorist Organization “Hamburg Cell”

**Figure 4:**
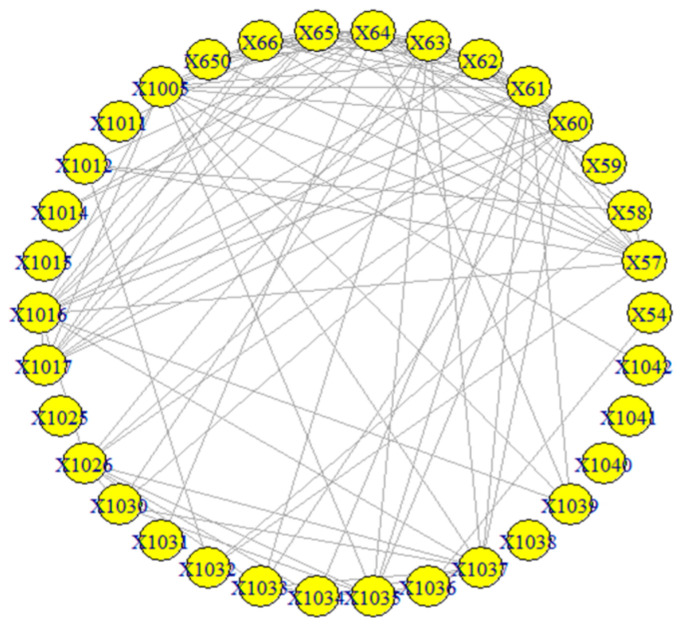
The network of “Hamburg Cell”.

The visualization of the overall network over the period 1985–2006 is presented in Figure 4.

**Figure 5:**
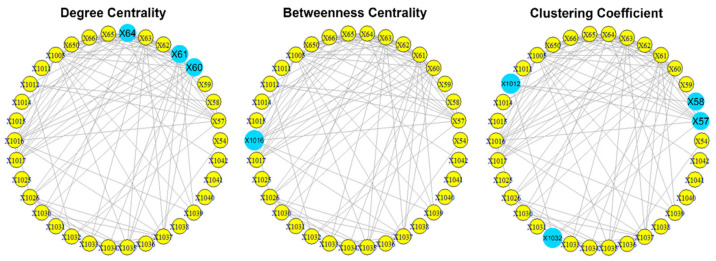
The overall protagonists of the “Hamburg Cell”. The protagonists are represented as larger light blue spheres. Most nodes have degree >0.3, but the nodes 64 (0.5), 61 (0.4) and 60 (0.4) stand out. Most nodes have low values (<0.1) as mediators, but the 1016 (0.2) stands out. Most nodes are not team players (clustering =0), but the nodes 58 (0.7), 1032 (0.7), 1012 (0.6) and 57 (0.4) stand out as team players.

**Figure 6:**
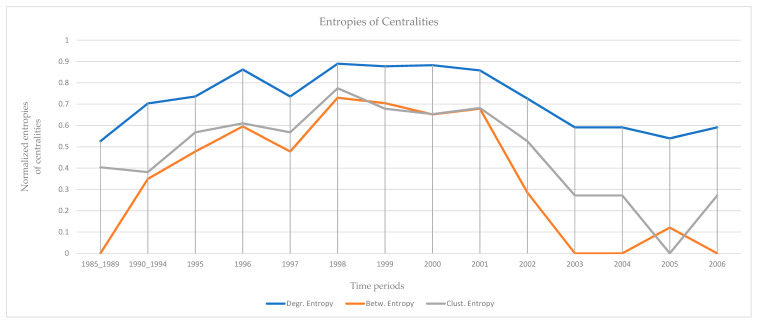
The evolution of normalized entropies. Two peaks appear in 1996 and 1998. All entropies decrease after 2001. The entropy of betweenness decreases faster. A plateau of high entropies appears in the period 1998 to 2001.

The results of the calculations of the centralities and the entropies are found in Supplementary Material 2. From the values of the centralities, the protagonists (high centrality nodes) for the period 1985–2006 (overall) are identified and presented in Figure 5. The protagonists for each time period are presented in Table 2. The evolution of the entropies of the centralities is presented in Figure 6.

### 4.3. Terrorist Organization “Al-Qaeda Section of Madrid”

**Figure 7:**
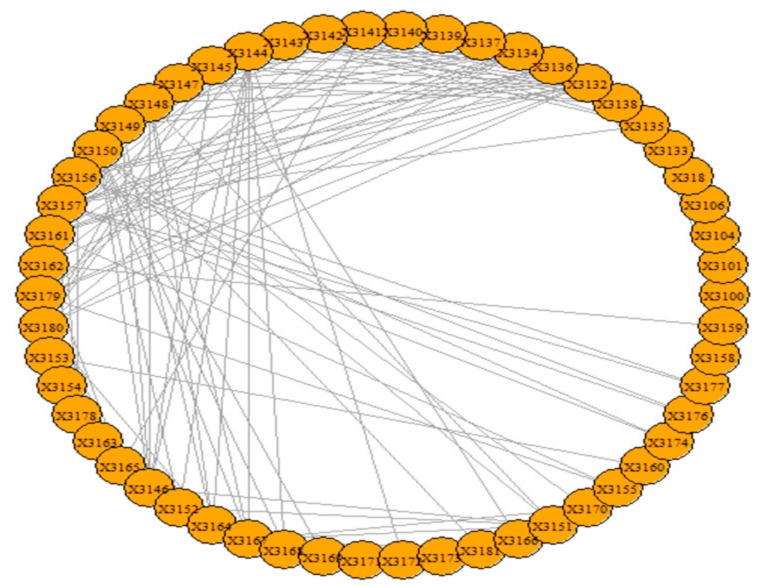
The network of the “al-Qaeda section of Madrid”.

The visualization of the overall network over the period 1985–2006 is presented in Figure 7.

**Figure 8:**
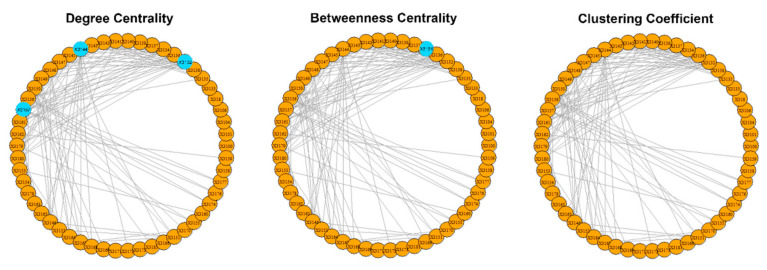
The overall protagonists of the “al-Qaeda section of Madrid”. The protagonists are represented as larger light blue spheres. Most nodes have degree >0.2, but the nodes 3132 (0.3), 3144 (0.3) and 3157 (0.3) stand out. Most nodes have low values (<0.1) as mediators, but the 3134 (0.2) stands out. All nodes are not team players (clustering =0).

**Figure 9:**
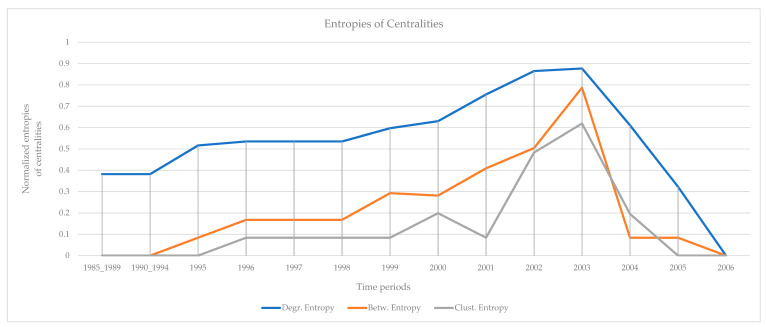
The evolution of normalized entropies. A sharp peak appears in 2003. All entropies decrease after 2003. The entropy of betweenness decreases faster.

The results of the calculations of the centralities and the entropies are found in Supplementary Material 3. From the values of the centralities, the protagonists (high centrality nodes) for the period 1985–2006 (overall) are identified and presented in Figure 8. The protagonists for each time period are presented in Table 3. The evolution of the entropies of the centralities is presented in Figure 9.

### 4.4. Terrorist Organization “Jamaah Islamiah Section of Philippines”

**Figure 10:**
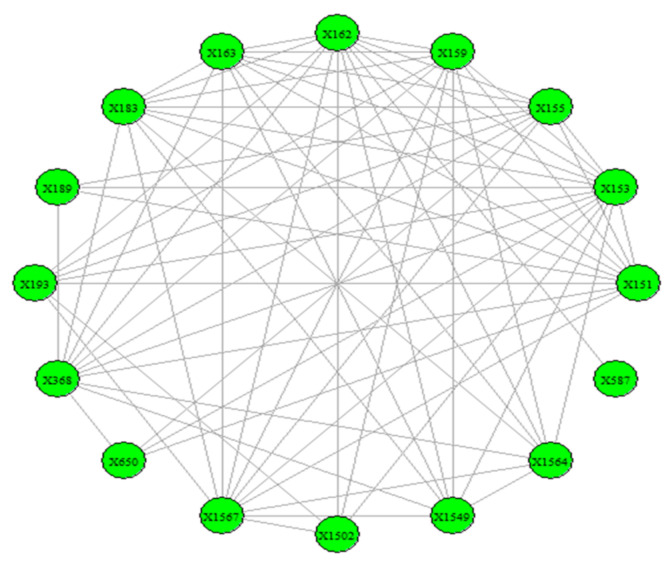
The network of the “Jamaah Islamiah section of Philippines”.

The visualization of the overall picture of the network 1985–2007 is presented in Figure 10.

**Figure 11:**
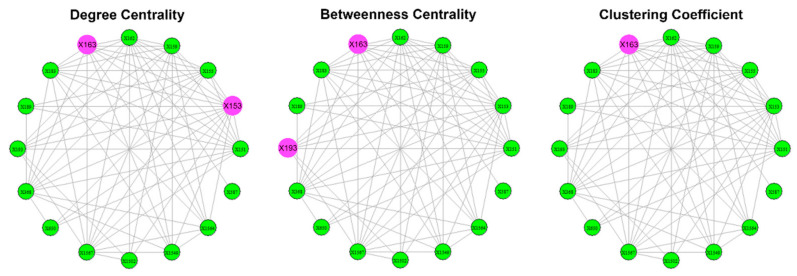
The overall protagonists of the “Jamaah Islamiah section of Philippines”. The protagonists are represented as larger pink spheres. Most nodes have high degree (≥0.7), but the 153 (0.9) and 163 (0.8) stand out. Most nodes have low values (≤0.1) as mediators but the nodes 193 (0.3) and 163 (0.2) stand out. Most nodes are team players (clustering ≥0.4), but the node 163 (1) stands out as team player.

**Figure 12:**
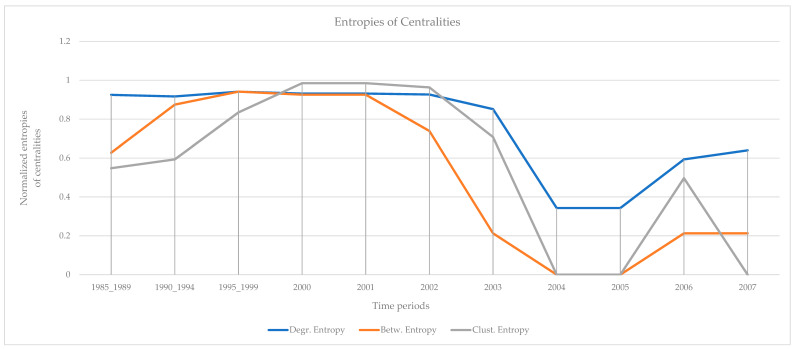
The evolution of normalized entropies. There is a high plateau of the values of entropies from 2000 to 2001. Afterwards, all entropies decrease for the next two years, and then, they increase to some extend after 2005. The entropy of betweenness decreases faster.

The results of the calculations of the centralities and the entropies are found in Supplementary Material 4. From the values of the centralities, the protagonists (high centrality nodes) for the period 1985–2007 (overall) are identified and presented in Figure 11. The protagonists for each time period are presented in Table 4. The evolution of the entropies of the centralities is presented in Figure 12.

## 5. Discussion

### 5.1. Different Roles of the Members of Terrorist Organizations

The different roles of the members of all four terrorist organizations (“Jamaah Islamiah section of Indonesia”, “Hamburg Cell”, “al-Qaeda section of Madrid”, “Jamaah Islamiah section of Philippines”) are clearly recognized from the values of the selected centralities. About 5–10% protagonists (nodes with high centralities) stand out in the four overall networks (Figure 2, Figure 5, Figure 8 and Figure 11). The members assume different roles in the time periods studied (Table 1, Table 2, Table 3 and Table 4). The mediators of the four organizations share a common temporal pattern (Table 1, Table 2, Table 3 and Table 4): Betweenness is decreasing to zero after a certain point in time. The rapid fall of betweenness centrality occurs shortly after a significant terrorist action [66,68,70,71,72,73]. An exception appears in the “Jamaah Islamiah section of Philippines” organization, as the mediators resume some value after the rapid fall (Table 4). This re-emergence of mediators coincides with their attempt to start another terrorist action [70].

“Jamaah Islamiah section of Indonesia” (Table 1): From 1990 to 2001 there is no division of roles among the members of the organization. From 2002 to 2006 the key nodes acquire higher values and new roles emerge. From 2005 to 2006 centralities decrease. Node 1506 appears in 2005 as a teamworking node receiving the highest value of all time periods. Node 0177 appears as teamworking node in 2003, and in the years 2004–2006, it appears as a high degree node, taking the highest degree in 2004 and at the same time being the only mediator. Node 1580 attracts the greatest interest, taking action for the first time in the period 1990–1994 as a high degree node, a quality that it maintains until 2001 with a small value but stable (0.1). The degree of node 1580 increases seven times (0.7) in 2002 assuming at the same time the role of mediator (0.3). Afterwards (2003–2005), node 1580 “disappears” as a central node, to reappear in 2006 as a teamworking node with the highest score (1.0). Nodes 1580 and 1595 share a common course from the first observation of the organization until 2001. However, node 1595 acquires the highest degree (1.0) in the year 2002 and afterwards “disappears” as a central node. It is remarkable that mediators in the organization appear only in the period 2002–2004.

“Hamburg Cell” (Table 2): In all time periods, the central nodes are strong team workers (clustering higher than 0.5) and lower degree values. This difference demonstrates operations of small groups weakly interconnected. In the periods 1985–1989 and 2002–2006, no high degree nodes and mediators appear, while in the period 1998 to 2002, new key players appear. Nodes 58, 1012 and 1032 share a common course in the periods 1985–1997 and 2002–2006 being also strong team workers (clustering higher than 0.5) with high values as (≥0.5) teamworking nodes. Node 57 is changing roles over the years. In the period 1985–1989 node 57 appears as a teamworking node, in the years 1990–1995 and 1997 as a high degree, while in the year 1996 as a mediator. Despite the emergence and disappearance of new nodes key players, node 60 is active in the organization continuously from 1995 to 2001.

“Al-Qaeda section of Madrid” (Table 3): In almost all time periods, many teamworking nodes appear. High degree nodes appear only in the period 2002–2003. No mediator appears except node 3157 in the year 2003. Node 3157 is the only teamworking node with high degree. Nodes 3155, 3161 and 3179 appear only in 2004 as the only teamworking central nodes with no role before or afterwards.

“Jamaah Islamiah section of Philippines” (Table 4): The network displays a diversification of roles in the periods 1985–2003 and 2006–2007, while in the years 2004 and 2005 no central node appears. In the period 1985–1989, node 155 has all three roles, node 151 appears as mediator with high degree, and node 162 appears only as a teamworking node with the highest clustering (0.1). The roles of three nodes have interesting development. Node 162 is only the only teamworking node from 1985 to 2003. Node 155 acquires various roles from 1985 to 2003. Node 151 acquires high degree and mediator roles from 1985 until 1999, then disappears as key player and reappears in 2002 as a mediator. Afterwards, node 151 disappears again and reappears in the period 2006–2007 as a high degree teamworking node.

### 5.2. Early Signs of Impending Terrorist Acts

An early sign of impending terrorist acts for all four terrorist organizations (“Jamaah Islamiah section of Indonesia”, “Hamburg Cell”, “al-Qaeda section of Madrid”, “Jamaah Islamiah section of Philippines”) is clearly recognized from the evolution of the values of the entropies of the selected centralities (Figure 3, Figure 6, Figure 9 and Figure 12). The entropies are increasing up to a certain point in time, and then, rapid decrease follows. This indicates that in periods of high entropy, many members acquire roles, while the roles are reserved for a few members only in periods of low entropy. These results are interpreted from real terrorist events:

“Jamaah Islamiah section of Indonesia”: The entropies of the centralities increase after 2002 and peak in 2004 (Figure 3), when the organization carries out its top strikes (2002 Bali, 2003 Marriott hotel, 2004 Australian embassy) [71,72,73].

“Hamburg Cell”: Two peaks of the entropies of the centralities appear in 1996 and 1998. A plateau of high entropies appears in the period 1998 to 2001. All entropies decrease rapidly after 2001 (Figure 6). The first peak (1996) coincides with the original planning of the attack on the twin towers (suggestion of Khalid Shaikh Mohammed to Bin Laden). The second peak (1998) coincides with the relocation to Hamburg of Mohamed Atta, Marwan al-Shehhi, and Ramzi bin al-Shibh, who were three of the hijackers on 9/11 (twin towers). The rapid decrease immediately after 2001, occurs immediately after the events of 9/11, in which members of the “Hamburg Cell” actively participated [66].

“Al-Qaeda section of Madrid”: The entropies of the centralities peak in 2003 (Figure 9), just before the major terrorist attack on the Madrid train in early 2004 [68].

“Jamaah Islamiah section of Philippines”: There is a high plateau of the values of entropies from 2000 to 2001. Afterwards, all entropies decrease for the next two years, and then, they increase to some extend after 2005 (Figure 12). The big bombing events happened in the change of the year 2000, immediately after the fall of entropies. The increase of the entropies after 2005 coincides with their attempt to start another terrorist action [70].

## 6. Conclusions

The research questions Q1 and Q2 have been addressed as follows:

The different roles of the members are clearly recognized from the values of the selected centralities (Section 5.1). An early sign of impending terrorist acts is the evolutionary pattern of the values of the entropies of the selected centralities (Section 5.2). These results have been confirmed (Section 5) by the real data (Section 3) from four real terrorist organizations in different countries.

Monitoring the three centralities (degree, betweenness, clustering) enables the law enforcement authorities to identify the roles of the members of terrorist organizations as the members with high centrality. Restricting the observation to the members with high centralities implies effective cost reduction. The recognition of distinct roles is one of the necessary requirements for the characterization of an organization as criminal—terrorist by the European Union [1,2].

Monitoring the evolution of entropies of the selected centralities (degree, betweenness, clustering) provides an early sign of impending terrorist acts. The observation of high entropies of the centralities is clearly an early sign, as in periods of high entropy, many members of the organization acquire roles in the network. This is clearly an additional input to law enforcement authorities for the prevention and suppression of terrorist strikes. In periods of high entropy, the authorities should be in high readiness. Law enforcement authorities have data from daily monitoring; therefore, they are able to assess events with much finer resolution, compared with the annual data used in this work, and draw more accurate conclusions. However, the methodology of the analysis is the same. Finally, it is tempting to observe the qualitative analogy of the temporal evolution of entropies (Figure 3, Figure 6, Figure 9 and Figure 12) with the evolution of readiness potentials [74].

## Figures and Tables

**Table 1:** Central nodes for each time period. The values of the centralities are indicated in parentheses. No value means that the centrality of all nodes v ≤is less than 0.09.

		Central Nodes		
		High Degree	Mediators	Teamworking Nodes
Periods	1985–1989	-	-	-
	1990–1994	**1580** (0.1)	-	-
		**1595** (0.1)		
	1995–1999	**1556** (0.1)	-	-
		**1580** (0.1)		
		**1595** (0.1)		
	2000	**1556** (0.1)	-	-
		**1561** (0.1)		
		**1562** (0.1)		
		**1570** (0.1)		
		**1580** (0.1)		
		**1595** (0.1)		
	2001	**1556** (0.1)	-	-
		**1561** (0.1)		
		**1562** (0.1)		
		**1570** (0.1)		
		**1580** (0.1)		
		**1582** (0.1)		
		**1590** (0.1)		
		**1595** (0.1)		
	2002	1556 (0.7)	1509 (0.2)	-
		1561 (0.5)		
		1562 (0.7)	1556 (0.2)	
		1580 (0.7)	**1580** (0.3)	
		1582 (0.7)	1582 (0.2)	
		1590 (0.7)		
		**1595** (1.0)		
	2003	**1579** (0.2)	1553 (0.2)	**0177** (0.6)
			**1556** (0.3)	**1574** (0.6)
			**1561** (0.3)	**1579** (0.6)
			**1590** (0.3)	
	2004	**177** (0.7)	**0177** (0.3)	**0801** (0.6)
		1579 (0.5)		**0802** (0.6)
				**1507** (0.6)
		1590 (0.5)		**1534** (0.6)
				**1563** (0.6)
				**1574** (0.6)
	2005	**177** (0.2)	-	**801** (1.0)
				1506 (0.8)
		**1562** (0.2)		**1574** (1.0)
	2006	**800** (0.1)	-	177 (0.3)
				0800 (0.3)
		**177** (0.1)		**1580** (1.0)
	2007		-	-

**Table 2:** Central nodes for each time period. The values of the centralities are indicated in parentheses. No value means that the centrality of all nodes v ≤is less than 0.09.

		Central Nodes		
		High Degree	Mediators	Teamworking Nodes
Periods	1985–1989	-	-	57 (0.6)
				**58** (0.7)
				1012 (0.6)
				**1032** (0.7)
	1990–1994	**57** (0.1)	-	**58** (0.7)
				1012 (0.6)
				**1032** (0.7)
	1995	**57** (0.1)	-	**58** (0.7)
		**60** (0.1)		1012 (0.6)
		**61** (0.1)		1030 (0.6)
		**63** (0.1)		**1032** (0.7)
				1035 (0.6)
	1996	**1026** (0.2)	**1026** (0.1) **57** (0.1) **61** (0.1)	**58** (0.7)
		**1035** (0.2)		1012 (0.6)
		**1037** (0.2)		1030 (0.6)
		**60** (0.2)		**1032** (0.7)
		**63** (0.2)		1033 (0.6)
	1997	**57** (0.1)	-	**58** (0.7)
		**60** (0.1)		1012 (0.6)
		**61** (0.1)		1030 (0.6)
		**63** (0.1)		**1032** (0.7)
				1035 (0.6)
	1998	**61** (0.4)	60 (0.1)	**1014** (0.7)
			1016 (0.1)	
		**64** (0.4)	64 (0.1)	**1017** (0.7)
			**65** (0.2)	
	1999	**64** (0.5)	**64** (0.1)	60 (0.6)
			**65** (0.1)	**1017** (0.7)
	2000	**60** (0.3)	**65** (0.1)	**60** (0.6)
		**61** (0.3)		
		**62** (0.3)		**66** (0.6)
		**64** (0.3)		
		**65** (0.3)		**1017** (0.6)
		**1005** (0.3)		
	2001	**60** (0.3)	**61** (0.1)	63 (0.6)
		**61** (0.3)		1017 (0.6)
		**62** (0.3)		**1039** (0.7)
		**64** (0.3)		
	2002	-	-	**64** (0.7)
				**1005** (0.7)
	2003	-	-	**58** (0.5)
				**1012** (0.5)
				**1032** (0.5)
	2004	-	-	**58** (0.5)
				**1012** (0.5)
				**1032** (0.5)
	2005	-	-	-
	2006	-	-	**58** (0.5)
				**1012** (0.5)
				**1032** (0.5)

**Table 3:** Central nodes for each time period. The values of the centralities are indicated in parentheses. No value means that the centrality of all nodes v ≤is less than 0.09.

		Central Nodes		
		High Degree	Mediators	Teamworking Nodes
Periods	1985–1989	-	-	-
	1990–1994	-	-	-
	1995	-	-	-
	1996	-	-	**3100** (0.1)
	1997	-	-	**3100** (0.1)
	1998	-	-	**3100** (0.1)
	1999	-	-	**3100** (0.1)
	2000	-	-	318 (0.8)
				3100 (0.1)
				**3104** (1.0)
	2001	**-**	-	**3100** (0.1)
	2002	**3132** (0.2)	-	**3135** (1.0)
				3138 (0.6)
				3140 (0.6)
				3148 (0.5)
		**3136** (0.2)		3149 (0.5)
				3156 (0.8)
				3157 (0.5)
				**3167** (1.0)
	2003	3132 (0.2)	**3157** (0.1)	3134 (0.5)
		3134 (0.2)		**3146** (1.0)
		**3144** (0.3)		**3147** (1.0)
		3145 (0.2)		3148 (0.6)
		3147 (0.2)		3149 (0.5)
		3148 (0.2)		3151 (0.6)
		3149 (0.2)		3156 (0.6)
		3150 (0.2)		3157 (0.5)
		3157 (0.2)		3165 (0.8)
				**3167** (1.0)
	2004	-	-	3155 (0.6)
				3161 (0.6)
				3179 (0.6)
	2005	-	-	-
	2006		-	-

**Table 4:** Central nodes for each time period. The values of the centralities are indicated in parentheses. No value means that the centrality of all nodes v ≤is less than 0.09.

		Central Nodes		
		High Degree	Mediators	Teamworking Nodes
Periods	1985–1989	**151** (0.4)	151 (0.1)	**155** (1.0)
		**155** (0.4)	**155** (0.2)	**162** (1.0)
	1990–1994	151 (0.4)	**368** (0.2)	**162** (1.0)
		153 (0.4)		
		155 (0.4)		
		159 (0.4)		
		163 (0.4)		
		**368** (0.5)		
		1564 (0.4)		
	1995–1999	**151** (0.6)	**151** (0.1)	155 (0.6)
		**153** (0.6)	**155** (0.1)	**162** (0.7)
		**155** (0.6)	**159** (0.1)	
		**163** (0.6)	**163** (0.1)	**183** (0.7)650 (0.6)
		**368** (0.6)	**193** (0.1)	
		**183** (0.6)	**368** (0.1)	
	2000	**153** (0.9)	**155** (0.1)	**162** (0.6)
				**189** (0.6)
		163 (0.8)	**163** (0.1)	**650** (0.6)
			**193** (0.1)	**1502** (0.6)
				**1567** (0.6)
	2001	**153** (0.9)	**155** (0.1)	**162** (0.6)
				**189** (0.6)
		163 (0.8)	**163** (0.1)	**650** (0.6)
			**193** (0.1)	**1502** (0.6)
				**1567** (0.6)
	2002	**153** (0.9)	151 (0.1)	**155** (0.7)
				162 (0.6)
			**155** (0.2)	189 (0.6)
		163 (0.8)		650 (0.6)
			163 (0.1)	1502 (0.6)
				1567 (0.6)
	2003	**153** (0.4)	**153** (0.1)	**155** (0.7)
				162 (0.6)
				189 (0.6)
				650 (0.6)
				1502 (0.6)
				1567 (0.6)
	2004	-	-	-
	2005	-	-	-
	2006	**151** (0.2)	-	151 (0.3)
		159 (0.1)		159 (0.5)
		1549 (0.1)		**1549** (1.0)
	2007	**151** (0.2)	-	-

## Data Availability

The data used in this study have been collected and published -freely for academic and research use- by the following organizations: 1. John Jay & ARTIS Transnational Terrorism Database (JJATT). [http://doitapps.jjay.cuny.edu/jjatt/index.php] (accessed on 1 August 2021). 2. Center for Computational Analysis of Social and Organizational Systems (CASOS) at Carnegie Mellon University. [http://www.casos.cs.cmu.edu/tools/datasets/external/index.php?]. (accessed on 1 August 2021). The humans depicted in the data are represented by code numbers. The number-human correspondence is not provided to the users of the data.

## Supplementary Materials

The following are available online at https://www.mdpi.com/article/10.3390/e23101334/s1, Supplementary Material S1: The results of the calculations of the centralities and the entropies of the “Jamaah Islamiah section of Indonesia”. Supplementary Material S2: The results of the calculations of the centralities and the entropies of the “Hamburg Cell”. Supplementary Material S3: The results of the calculations of the centralities and the entropies of the “Al-Qaeda section of Madrid”. Supplementary Material S4: The results of the calculations of the centralities and the entropies of the “Jamaah Islamiah section of Philippines”.
